# Boredom in Adolescence: Validation of the Italian Version of the Multidimensional State Boredom Scale (MSBS) in Adolescents

**DOI:** 10.3390/children8040314

**Published:** 2021-04-20

**Authors:** Andrea Spoto, Sara Iannattone, Perla Valentini, Alessia Raffagnato, Marina Miscioscia, Michela Gatta

**Affiliations:** 1 Department of General Psychology, University of Padua, 35131 Padua, Italy; andrea.spoto@unipd.it; 2 Department of Women’s and Children’s Health, Padua University Hospital, 35128 Padua, Italy; alessiaraffagnato@gmail.com (A.R.); marina.miscioscia@unipd.it (M.M.); michela.gatta@unipd.it (M.G.); 3 Department of Communication Sciences, Humanistic and International Studies: History, Culture, Languages, Literature, Arts, Media, University of Urbino ‘Carlo Bo’, 61029 Urbino, Italy; p.valentini4@campus.uniurb.it

**Keywords:** adolescence, assessment, boredom, confirmatory factor analysis, mental health, risk factors

## Abstract

Boredom in adolescence is often underestimated, although it may be the sign of a profound unease or be associated with psychological disorders. Given the complexity of the construct of boredom and its increasing prevalence among adolescents in recent years, the present study aimed to validate the factorial structure of the Italian version of the Multidimensional State Boredom Scale (MSBS) in adolescents using a cross-validation approach. The study involved 272 students (33.8% males, 66.2% females) aged 14–19 (M = 15.9, SD = 1.38) living in northern and central Italy. In addition to the MSBS, the Symptoms Checklist 90-R (SCL 90-R) and the Children’s Depression Inventory (CDI) were administered. Exploratory and confirmatory factor analyses validated a 23-item structure of the MSBS, comprising five correlated factors. The tool showed a good internal consistency for these factors and a good convergent and factor validity. The MSBS consequently seems a valid and reliable method for assessing boredom in adolescence. The cut-off for the total score that could pinpoint cases posing a potential clinical risk was 88. A weak correlation was found between the total level of boredom and the daily Internet usage, while no relationship emerged between boredom and age, gender, and grades. Since excessive levels of boredom may conceal a general unease that could develop into structured psychological disorders, the value of the MSBS lies in enabling us to identify in advance adolescents at potential clinical risk.

## 1. Introduction

### 1.1. General Considerations and Recent Explanatory Models of Boredom

Boredom is a common affective state that may be experienced by people of any age, and that is why it is often considered a normal feature of daily life [1]. There is currently no single, shared definition of boredom. According to various theories, it has been conceived as a characteristic of human nature, a temperamental trait, a reaction to monotonous environmental conditions, or the sign or symptom of a specific disorder [2]. The only aspect on which authors all seem to agree is that this is an unpleasant affect characterized by a feeling of wanting something without knowing what and being unable to find something sufficiently satisfactory to do [3]. Moreover, Mills and Christoff [4] stated that boredom represents a challenge to scientific research because of its complexity. The authors also pointed out the importance of appreciating this experience for its temporal instability and of better investigating its dynamics.

The current literature in the field of boredom has rejected the historical theories which considered only the negative aspects of this affect. Indeed, recent conceptualizations have underlined the functionality of boredom since it indicates that a situation is unsatisfactory (e.g., [5,6,7]) and motivates people to explore more interesting behavioral alternatives (e.g., [8,9]). Specifically, van Tilburg and Igou [10,11] suggested that boredom arises when one’s goal is low in meaning; in this case, boredom acts as an important signal which promotes the re-engagement in activities that are coherent with one’s interests, thus reestablishing a sense of meaningfulness.

Westgate and Wilson [12] proposed a comprehensive explanatory model of boredom—the Meaning and Attentional Component (MAC) model—according to which boredom derives from an incongruence between cognitive demands and available mental resources (attentional component) and/or an incongruence between activities and valued goals (meaning component). Consequently, people become bored when they are unable to pay attention to an activity and/or consider the activity meaningless.

Finally, the relevance of boredom has been recently emphasized by Martarelli et al. [13], who studied boredom proneness in homeschoolers during the COVID-19 pandemic. The authors found that students with high boredom proneness perceived homeschooling as more difficult, thus resulting in those students being more disadvantaged in this kind of setting. Therefore, Martarelli et al. pointed out the importance of taking into consideration boredom in the context of homeschooling in order to prevent its detrimental effects on learning. On the basis of the current literature, the authors proposed some possible solutions to reduce boredom in a homeschooling setting, such as removing general distractions from the environment, offering meaningful activities, and helping students to identify and use boredom in an adaptative way.

### 1.2. Adolescent Boredom: Specificity, at Risk Behaviors and Psychological Disorders

Given the complexity and the importance of the construct of boredom, we take a closer look at its features, especially in adolescence, when an individual’s emotional balance may already be precarious. Although this affective state is quite common among teenagers [14], it is not easy to study its peculiarities in this developmental age group. This is partly because of the various changes associated with adolescence per se, which contribute to making boredom and its manifestations even more multifaceted [15].

Adolescence coincides with an increase in the speed and efficiency of an individual’s basic cognitive processes, and Keating [16] suggested that adolescents might consequently perceive many tasks as repetitive, making them feel scarcely motivated and bored. Hamilton [17] associated boredom with temperamental traits: teenagers who report being more inclined to sensation seeking also tend to have more difficulty finding something interesting to do and are more likely to become bored.

In the psychosocial sphere, some authors claim that adolescents are more likely to become bored when their social surroundings—including their reference adults [15], school [18] and peers [19,20], and their environment [21]—fail to offer appropriate leisure activities. The problem sometimes lies in the adolescents themselves lacking sufficient personal resources to engage in satisfying activities [2]. Within the family, one issue that has been associated with higher levels of adolescent boredom is excessive parental control. Boredom can be the result of an adolescent’s attempt to resist limitations imposed by an adult [15,20].

According to a study by Weybright et al. [22], boredom in adolescence has been on the rise in recent years. The authors suggested that this might stem from our changing times and especially the increasingly intensive use of the Internet and technology. This idea was first advanced by Biolcati et al. [23], who examined studies on the association between boredom in adolescence and Internet usage [24,25,26]. Biolcati et al. found that teenagers more likely to become bored were those who made more use of technology and engaged less in other activities (such as sports), and this predisposed them to forms of Internet dependence. A study by Stockdale and Coyne [27] on a sample of adolescents showed that the most common reason for their use of social networks was to relieve boredom. Griffiths [28] also found that a frequent tendency to escape into the online world to cope with negative states of mind such as boredom raises the risk of Internet dependence.

Other researchers identified an association between boredom in leisure time and the risk of developing a dependence on alcohol or drugs. Young people in the habit of using such substances have a greater tendency to become bored because they are more inclined towards sensation seeking, always wanting to try new emotions [29]. Biolcati et al. [30] subsequently demonstrated that a predisposition to boredom facilitates the psychological expectation that alcohol can alleviate suffering, thereby promoting its consumption.

Boredom has revealed an important role in adolescent delinquency as well. Newberry and Duncan [31] found that one of the reasons often given for delinquent behavior in adolescent age is to “pass the time,” when teenagers lack the personal resources to deal with their boredom. Spaeth et al. [2] suggested that boredom and adolescent delinquency have mutually instigating effects: delinquent behavior risks leading to social marginalization, which tends to promote boredom, and deviant behavior may be used as a way to alleviate boredom.

A close relationship has been found between boredom proneness and depression, albeit these constructs are different in mood quality and intensity [32]. Specifically, a study by Spaeth et al. [2] shed light on the association between boredom in leisure time and depression in adolescents. Feeling depressed and the type of behavior this causes (social withdrawal, lack of activities and intrinsic motivation) can interfere with adolescents’ active search for pleasant experiences in their free time, amplifying their boredom. At the same time, boredom could facilitate a state of depression by making adolescents find nothing sufficiently gratifying.

### 1.3. Tools for Measuring Boredom

The main scales currently available for measuring boredom only assess this construct in certain situations, such as: in an individual’s spare time (Leisure Boredom Scale [33]; Free Time Boredom Scale [34]), at work (Job Boredom Scale [35]), at school (Academic Boredom Scale [36]), or in the sexual sphere (Sexual Boredom Scale [37]) [3].

One full-scale tool for directly measuring boredom is the Boredom Proneness Scale (BPS, [38]), consisting of 28 items. This questionnaire was designed to identify trait boredom, which means a tendency to become bored [3]. Since its factorial structure was found to be highly unstable (e.g., [39,40]), Vodanovich et al. [41] validated a 12-item version of the BPS (BPS-SF) with two factors. Subsequently, Struck et al. [42] proposed the Short Boredom Proneness Scale (SBPS), composed of eight items and one factor. The SBPS was recently validated in German [43] and Chinese [44] samples, thus supporting the cross-cultural consistency of the one-factor model of boredom proneness.

Nevertheless, the full BPS and its different short versions do not measure state boredom, which is the experience of being bored in a given moment. Judging the lack of tools for identifying state boredom as a major shortcoming, Fahlman et al. [3] developed their Multidimensional State Boredom Scale (MSBS). The authors emphasized the importance of measuring state boredom because this enables us to investigate the potential causes and consequences of boredom in general in greater depth. Unlike trait boredom, which is an abstraction, state boredom is a concrete experience. According to the authors of the MSBS, we cannot tell whether individuals are liable to boredom without first establishing whether they have been bored in any given moment. They claimed that the MSBS can serve as a fundamental tool for further analyzing both state boredom and a predisposition to boredom, so that we can speak of boredom as a whole, without distinguishing between these two components [3].

**Figure 1:**
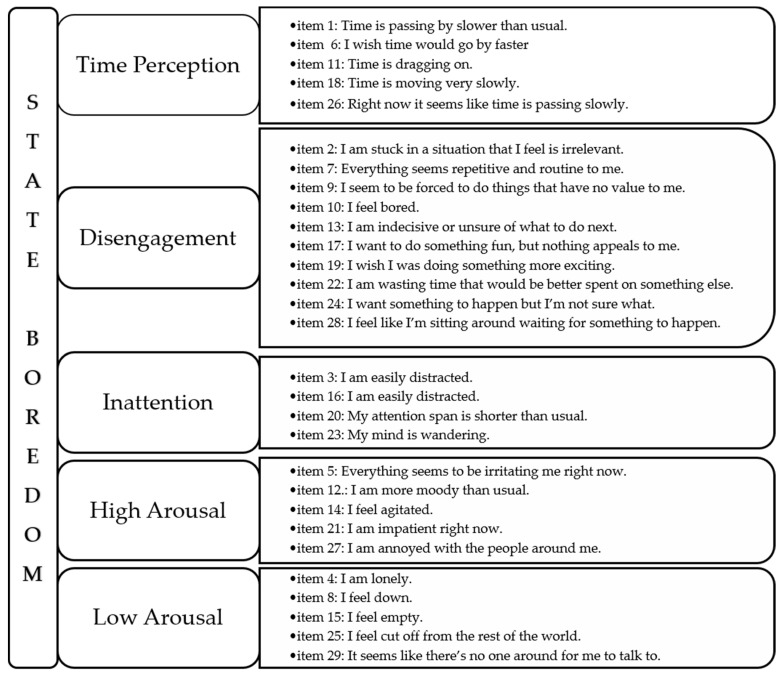
Factor structure and item grouping in the original version of the Multidimensional State Boredom Scale (MSBS). Notes: TP, time perception; DIS, disengagement; INA, inattention; HA, high arousal; LA, low arousal.

This scale consists of 29 items, and respondents give their answers on a seven-point Likert scale (from 1 = strongly disagree to 7 = strongly agree). The items are divided into five factors: (a) time perception (TP), which describes the slow passage of time; (b) disengagement (DIS), regarding a lack of involvement; (c) inattention (INA), or difficulty focusing attention on events; (d) high arousal (HA), which concerns the negative effects of an excessively high arousal; and (e) low arousal (LA), which covers the experiences and behavior attributable to an excessively low arousal. Scores obtained for these five factors are combined to obtain an overall boredom score (Figure 1). This factorial structure has been confirmed in Chinese [45], Australian [46], Turkish [47], Spanish [48], and Italian [49] adult samples.

Moreover, two shorter versions of the MSBS have been developed: the MSBS-SF [50], which is unidimensional and composed of 8 items; and the MSBS-15 [51], which is multidimensional and has 15 items. Both have good psychometric properties, and their performances were found to be similar to those of the original MSBS.

As concerns the adolescent population, given that high levels of boredom may pose some inherent risks in teenagers, a scale to assess this affect is certainly useful for both clinical and research purposes. For this reason, Donati et al. [52] recently validated the MSBS-SF in an Italian sample. This study confirmed the one-dimensionality of the questionnaire and showed that it is reliable in measuring state boredom among both male and female adolescents.

However, in light of the complexity and the multidimensional nature of the construct of boredom, it seems also important to further investigate all its specific sub-components. Therefore, the aim of the present work is to apply the full Italian version of the MSBS, previously validated in Italian adults by Craparo et al. [49], to adolescents. Considering the paucity of recent studies analyzing the features of adolescent boredom in depth, our study might represent a significant contribution to the literature in this field.

## 2. Materials and Methods

### 2.1. Participants and Procedure

Data were collected from December 2018 to January 2020 at three upper secondary schools (grades 9 and higher) in northern and central Italy. The students’ participation in this study was approved by the school directors, and the students and both their parents signed to their informed consent. Moreover, this study was part of a broader screening project about risk factors in adolescence, conducted in accordance with the recommendations of the Declaration of Helsinki and approved by the local ethical committee (CESU, October 2019, prot.23).

The procedure was explained to the classes during school hours at each school, and anonymity was guaranteed; then, the same questionnaires were distributed to all participants (see list below). They first completed an information sheet to collect general information for the purposes of the study (e.g., number of out-of-school activities, amount of time spent each day on the Internet, average school marks, etc.). The questionnaire was administered collectively, and the time taken to complete the protocol in each class was approximately 30 min.

The sample consisted of 272 adolescents: 92 males (33.8%) and 180 females (66.2%), from 14 to 19 years of age (M = 15.9, SD = 1.38). Specifically, the vast majority of the sample was composed of adolescents aged 14 to 17. Only 46 participants were 18 years old (i.e., 16.9% of the whole sample), and 4 were 19 years old (i.e., 1.5% of the whole sample).

### 2.2. Measures

**Multidimensional State Boredom Scale (MSBS).** In this study, we used the Italian version of the MSBS validated in a sample of adults by Craparo et al. [49]. It has the same factor structure as the original questionnaire (i.e., 29 items and 5 factors). The psychometric properties of the tool are good in both the original and Italian versions. As concerns the internal consistency of the former, Cronbach’s alpha is 0.94 for the total score and ranges from 0.80 and 0.88 for the single factor scores [3]; about the internal consistency of the latter, Cronbach’s alpha is 0.95 for the total score and ranges from 0.80 to 0.89 for the single factor scores [49].

**Symptom Checklist 90-R (SCL-90-R).** This is a self-administered questionnaire designed by Derogatis [53]. It consists of 90 items that respondents answer on a five-point Likert scale (from 0 = not at all to 4 = very much). This tool measures internalizing and externalizing symptoms and comprises nine scales [54]: depression (DEP); obsessive-compulsive disorder (O-C); somatization (SOM); interpersonal sensitivity (INT. SENS); anxiety (ANX); hostility (HOS); phobic anxiety (PHOB); paranoid ideation (PAR); and psychoticism (PSY). There are seven additional items (that are not part of the scales) for measuring problems of appetite and sleep-related issues. Combining the scores for each scale generates a total score (the Global Severity Index—GSI), which gives a general measure of the severity of an individual’s perceived psychological discomfort. As concerns the checklist’s psychometric properties, all the scales have a good internal consistency, with a Cronbach’s alpha in the range of 0.77 to 0.90. The Italian version of the checklist [54] used in the present study also achieves a satisfactory reliability, with a Cronbach’s alpha ranging between 0.68 and 0.87 for the single scales and reaching 0.97 for the GSI.

**Children’s Depression Inventory (CDI).** This is a self-report questionnaire for children and adolescents developed by Kovacs [55] to identify symptoms of depression in developmental age. It consists of 27 items and respondents choose which of several options best describes their thoughts and feelings in the previous two weeks. The tool has a good internal consistency, with Cronbach’s alpha proving acceptable in samples of both psychiatric patients (α = 0.86) and schoolchildren (α = 0.87) [55]. The Italian version of the questionnaire used in our study, developed by Camuffo et al. [56], has a good reliability too, with Cronbach’s alpha ranging between 0.69 and 0.76.

### 2.3. Data Analysis

Descriptive statistics and frequency tables were calculated first to ascertain the characteristics of the study population.

Then the sample was divided semi-randomly into two groups of 136 participants each in order to conduct a cross-validation study. The aim was to test the original factor structure of the MSBS when applied to the Italian adolescent population. The proportion of males and females in the two groups was the same as in the original sample.

An exploratory factor analysis (EFA) was conducted on one of the groups (*N* = 136). The Shapiro-Wilk’s test [57,58] was used to verify the multivariate normality of the data. The number of factors was then established using the extraction method based on parallel analysis with maximum likelihood and promax rotation. We examined whether the data matrix could be factorialized using Bartlett’s sphericity test [59] and the Kaiser-Meyer-Olkin (KMO) test for sampling adequacy [60]. Visual inspection of the scree-plot was adopted as a criterion to confirm the optimal number of factors. The factor structure that best fitted our data was established using the following fit indices: the Root Mean Square Error of Approximation (RMSEA, [61]); the Tucker-Lewis Index (TLI, [62]); the Bayesian Information Criterion (BIC, [63]); and the ratio of chi-square to degrees of freedom (χ²/df).

A confirmatory factor analysis (CFA) was conducted on the other group (*N* = 136), testing the best factorial model resulting from the previous EFA. The estimation method used was maximum likelihood, which is shown to be the most robust in case of deviation from multivariate normality (e.g., [64]). The following fit indices were considered to test the goodness of the model fit: the ratio of chi-square to degrees of freedom (χ²/df); the Comparative Fit Index (CFI, [65]); the TLI; the Standardized Root Mean Square Residual (SRMR, [66]); the RMSEA; Akaike’s Information Criterion (AIC, [67]); and the BIC. Values of the CFI and TLI near 0.95, values of the SRMR ≤ 0.08, values of the RMSEA ≤0.06 [68], and a χ²/df ratio < 3 (e.g., [69]) indicate an acceptable fit.

The internal consistency of each of the scales was assessed using McDonald’s omega (ω, [70]), item-rest correlation, and inter-item correlation.

Subsequently, the whole sample was used to test the convergent validity of the MSBS by calculating Pearson’s r correlations between the factors comprising the MSBS, the total score for the CDI, and the scores for interpersonal sensitivity, depression, and hostility scales, and the GSI for the SCL-90-R. The same correlations were also analyzed for males and females separately, in order to verify whether the relationship between boredom and the considered psychopathological features differed according to gender.

Then, the descriptive indexes of the resulting factors in the MSBS were calculated.

An analysis of the Receiver Operating Characteristic (ROC) curves was also conducted. This is a widely used method for identifying the optimal cut-off value of a test and for assessing its diagnostic accuracy [71]. The accuracy of a diagnostic test depends on its sensitivity (i.e., a measure of how well it can identify true positives) and specificity (i.e., a measure of how well it can identify true negatives). The ROC curve is generated by plotting sensitivity, calculated at every possible cut-off point, against 1-specificity. To obtain the best cut-off value of a test, the optimal balance between sensitivity and specificity must be struck. We used the ROC curves to establish a cut-off for the total MSBS score that would enable us to identify adolescents at higher clinical risk with the best levels of sensitivity and specificity. The criterion used in this analysis was the dichotomized average score for the GSI of the SCL-90-R, taken to be representative of a general sense of unease of clinical interest if >1 [53,72,73].

Furthermore, the relationships between boredom (total MSBS score) and age, daily Internet usage, grades, and gender were analyzed. Specifically, Pearson’s r correlations were conducted considering age, while Spearman’s rho (*ρ*) correlations considered Internet usage and grades. Finally, an independent sample t-test was run entering the participants’ gender as an independent variable.

The above-described analyses were conducted using the Jamovi 1.6.1 statistical software [74] and R 3.6.3 [75]. To be more specific, the Factor module in Jamovi [76,77] was used for the EFA, CFA, and reliability analyses, and the pROC package in R [78] to analyze the ROC curves.

## 3. Results

### 3.1. Description of the Sample

Regarding the participants’ Internet usage (including social networks, but not WhatsApp), 39.3% of the sample reported spending no more than two hours a day on Internet usage, 55.9% spent from 2 to 5 h a day, and 4.8% more than 5 h a day.

As for their average school marks, the students reported being below pass level in 16.9% of cases and at pass level in 34.9%, while 43.4% had good marks and 4.8% had excellent marks. In their spare time, 87.5% of participants engaged in one out-of-school activity, 11% in two, and 1.5% in more than two.

Concerning the parents’ formal education, 4.4% (of both mothers and fathers) did not answer the question. Of the mothers who did, 1.1% had reportedly completed primary school, 19.5% had finished middle school, 42.8% had a high-school diploma, and 26.8% had a university degree or postgraduate education (Ph.D., masters, or specialization courses). Of the fathers, 0.54% had completed primary school, 23.2% had finished middle school, 49.6% had a high-school diploma, and 22.4% had a university degree or higher qualification.

Finally, Table 1 shows the mean scores obtained on the CDI total score; the interpersonal sensitivity, depression, and hostility scales; and the GSI of the SCL-90-R.

### 3.2. Exploratory Factor Analysis (EFA)

The tests to ascertain the feasibility of factorializing our data matrix confirmed that it was amenable to EFA (KMO = 0.85; Bartlett’s sphericity test: χ² = 2,234, df = 406, *p* < 0.001). The overall Shapiro-Wilk’s test showed that our data significantly deviated from a multivariate normal distribution (W = 0.861, *p* < 0.001).

Visual inspection of the scree-plot resulting from the EFA suggested a solution with four factors. As recommended in the literature [79,80], further EFA were conducted, testing not only the model with the number of factors suggested by the scree plot but also models with one more or one less factor. In addition, the one-factor model was tested, too. As shown in Table 2, the model with five factors showed the best-fit indices overall, so this solution was selected.

It became clear from the last EFA that item 17 did not saturate on any of the factors, and item 27 saturated on both Factor 1 and Factor 3, so these two items were removed from the model. Table 3 shows the distribution of the items and the standardized saturations on the respective factors. Items 4 and 16 had saturations > 1. As Jöreskog [81] explained, this is because standardized saturations in EFA in which the factors are correlated (oblique rotation) are regression coefficients, not correlations, so it is acceptable for some parameters to be estimated at >1.

As for the distribution of the items among the factors, items 2, 13, 24, and 28 (originally part of disengagement) and item 23 (belonging to inattention) were found associated with other factors. To be specific, items 13 and 24 joined Factor 1, while items 2, 23, and 28 joined Factor 3. In the light of the items’ content and their new distribution amongst the factors, the factors in our five-factor model of the MSBS were named as follows:

- Factor 1: internalizing aspects (INT);
- Factor 2: time perception (TP);
- Factor 3: high arousal (HA);
- Factor 4: inattention (INA);
- Factor 5: disengagement (DIS).

### 3.3. Confirmatory Factor Analysis (CFA)

When a CFA was conducted on the second group to test the five-factor model of the MSBS (Model 1), it emerged that item 23 had a triple saturation, while item 2 had a non-significant saturation on its own factor. Another model was therefore tested (Model 2) after removing both these items. The CFA on Model 2 showed that items 4 and 25 had multiple saturations, so these two items were also excluded, and a third model was analyzed. Table 4 shows the fit indexes for the CFA. The final model (Model 3) revealed the best fit. The standardized and unstandardized saturations of the items on their respective factors are shown in Table 5.

As the covariances between the factors were all significant, ranging between 0.19 and 0.90, we were also able to consider the total score obtained with the MSBS. Consistently with the findings of the study conducted to validate the Italian version [49], the time perception factor presented the lowest covariances with the other factors, which ranged between 0.19 and 0.44.

Despite the removal of some items from the original model, the overall significance of the single factors remained the same, so the original names of these factors were retained. The final factor structure of the Italian version of the MSBS for adolescents was as follows:

- internalizing aspects (INT): items 8, 13, 15, 24, 29;
- time perception (TP): items 1, 6, 11, 18, 26;
- high arousal (HA): items 5, 12, 14, 21, 28;
- inattention (INA): items 3, 16, 20;
- disengagement (DIS): items 7, 9, 10, 19, 22.

This final 23-item version of the MSBS is presented in the Appendix A (English translation) and in the Appendix B (original Italian items). The descriptive indexes are shown in Table 6.

### 3.4. Internal Consistency of the Scales

Our reliability analysis adopting the new factor structure of the MSBS showed that McDonald’s ω coefficient was 0.90 overall, and the item-rest correlations varied between 0.32 and 0.65. For the single factors, McDonald’s ω coefficient ranged between 0.72 and 0.92 and the item-rest correlations between 0.41 and 0.86 (Table 7). The inter-item correlations ranged from 0.30 to 0.50 for internalizing aspects, from 0.45 to 0.85 for time perception, from 0.23 to 0.43 for high arousal, from 0.55 to 0.67 for inattention, and from 0.26 to 0.50 for disengagement. Taken together, these data were satisfactory and indicate a good reliability of the tool.

### 3.5. Convergent Validity

For the whole sample, Table 8 shows the values obtained for Pearson’s r correlations between the factors comprising the MSBS, the total CDI score, and the interpersonal sensitivity, depression, and hostility scales and GSI of the SCL-90-R. Clearly, the time perception factor does not correlate with any of the scales, while the inattention and disengagement factors show moderate correlations with all the scales except for interpersonal sensitivity and hostility, with which they correlate only weakly. As for the high arousal factor and the total MSBS score, the strongest correlations emerge with the total CDI score, the depression scale, and the GSI, while the correlations with the other scales are only moderate. Finally, the internalizing aspects factor correlates strongly with all the scales except hostility.

When males (*N* = 92) and females (*N* = 180) were considered separately, however, significant differences emerged in the correlations between total MSBS score and hostility (z = 2.059, *p* = 0.02, r_males_ = 0.48, r_females_ = 0.25) and between internalizing aspects and hostility (z = 1.846, *p* = 0.032, r_males_ = 0.45, r_females_ = 0.24).

### 3.6. Cut-off for the Total MSBS Score

The Area Under the Curve (AUC) of the ROC curve was 75.8% (95% confidence interval = 70–82%). Considering that a test with an AUC value between 70% and 90% presents moderate accuracy [71], our analysis indicated that the MSBS possessed moderate discriminative ability. To establish the optimal cut-off value for the total MSBS score that enabled us to discriminate between adolescents with and without signs of boredom of potential clinical relevance, we analyzed both specificity and sensitivity at each possible cut-off point. The analysis showed that a total MSBS score of 88 allowed us to optimize the median sensitivity and specificity of the scale at 70% and 71%, respectively, so this was the best cut-off score to pinpoint cases posing a potential clinical risk.

### 3.7. Relationship between Boredom and Age, Internet Usage, Grades, and Gender

A low correlation emerged only between the total MSBS score and daily Internet usage (*ρ* = 0.13, *p* = 0.035). The other analyses (Pearson’s r and Spearman’s rho correlations, and independent sample t-test) did not show any significant relationship between the total level of boredom and age (r = −0.049, *p* = 0.425), grades (*ρ* = −0.029, *p* = 0.636), and gender (t_270_ = −1.49, *p* = 0.137).

## 4. Discussion

The main aim of the present study was to validate the Italian version of the MSBS for use with adolescents, and our cross-validation study demonstrated that the original structure with five factors, and 23 of the original 29 items, showed an adequate fit with our data.

As concerns the distribution of the items among the factors envisaged in the MSBS, the time perception, inattention, and disengagement factors remained substantially the same in the present version as in the original MSBS, while some differences emerged for the low arousal and high arousal factors. To be specific, item 13 (“I am indecisive or unsure of what to do next”) and item 24 (“I want something to happen but I’m not sure what”), initially part of the disengagement factor, joined the items relating to low arousal. The content of the new factor thus created refers generally to signs of depression and anxiety, so we changed its name from low arousal to internalizing aspects. On the other hand, item 28 (“I feel like I’m sitting around waiting for something to happen”), which also belonged originally to disengagement, joined the items relating to high arousal. The content of item 28 concerns aspects of internal activation and agitation, consistent with the meaning of high arousal, so the name of this factor was left unchanged.

The five resulting factors correlated significantly with one another, so we were able to consider a total score for boredom, too. That said, time perception showed the weakest correlation with the other factors of the MSBS, as already seen in the study conducted to validate the Italian version of the tool [49]. Looking at the items belonging to the time perception factor, their content is clearly repetitive and very specific, focusing on the slow passage of time. This was confirmed by the internal consistency indexes, which are very high in absolute terms for time perception, whereas the items belonging to the other factors have a more varied content, investigating different facets of the same macro-construct of each factor. These general considerations might explain the weak association between time perception and the other factors in the questionnaire.

As concerns the psychometric properties of the MSBS, both the single factors and the total score showed a good internal consistency. This means that it can reliably measure both the overall experience of boredom in adolescence and single dimensions of this affect.

Our findings regarding the correlations between the MSBS and the other tools used to measure constructs associated with boredom in the literature (the CDI and the SCL-90-R scales for depression, interpersonal sensitivity, and hostility and the GSI) are generally satisfactory, supporting a good convergent validity of the MSBS. The only factor unassociated with the other measures was time perception. This may be because a distorted perception of the passage of time in adolescence is not strongly related to any psychopathological dimension, but rather to this developmental stage per se. On the other hand, the internalizing aspects factor correlates strongly with the total CDI score and with the interpersonal sensitivity and depression scales of the SCL-90-R, confirming its close association with internalizing problems. Finally, when we considered our sample as a whole, hostility emerged as the SCL-90-R scale that correlated the least with the factors of the MSBS. This finding could stem from the fact that boredom in adolescence is strongly associated with internalizing problems and with a general sense of unease rather than with aggressive thoughts and behavior or irritability. That said, when we grouped our sample by sex and compared males and females, there was a significant difference in the way the hostility scale correlated with the total MSBS score and the internalizing aspects factor. In short, we were unable to reach any final conclusions concerning the association between boredom and hostility because it may be sex related. This aspect will need to be further analyzed in future studies.

In previous reports, boredom in adolescence was associated with psychopathological issues such as depression (e.g., [2]), Internet dependence (e.g., [28]), binge drinking (e.g., [30]), substance use (e.g., [29]), and delinquency (e.g., [2]). It therefore seemed important to identify a cut-off in the total MSBS score for discriminating between individuals more or less exposed to a clinical risk. Judging from the data deriving from our ROC curve analysis, neither the sensitivity and specificity levels nor the area under the curve meets the criteria to support a strong discriminatory power of the MSBS, taking the GSI of the SCL-90-R for reference. These results could derive from the fact that, although boredom is sometimes associated with behavioral and psychological signs of unease, it is not a clinical condition in itself. Scores above the cut-off that we identified should therefore be interpreted as an indication not necessarily of a psychological disorder but rather of a malaise that is worthy of attention and may warrant monitoring. The value of a screening tool capable of identifying indicators of a degree of vulnerability lies in that the experience of psycho-emotional suffering may evolve into structured psychopathological issues under certain individual and environmental conditions that may pose a risk. In terms of primary and secondary prevention, it is important to distinguish situations in which boredom is a physiological and phase-specific feature of the adolescent crisis from those in which it is the outward sign of potentially psychopathological conditions.

Finally, about the relationship between boredom and age, daily Internet usage, grades, and gender, the only positive—albeit low—correlation emerged with the amount of time spent on the Internet. This result, in line with previous studies (e.g., [23]), suggests that adolescents who spend more time online are also more bored. From the clinical standpoint, these data should be carefully taken into mind with those psychological disorders characterized by inattention and/or impulsivity, given that boredom proneness was found to be associated with Internet addiction in adolescents with attention-deficit/hyperactivity disorder [82]. Nevertheless, this correlation is too weak to support a definitive conclusion regarding the association between boredom and Internet usage. 

As regards the relationship between boredom and gender, the existing literature presents contrasting results [83]: for instance, Newberry and Duncan [31] showed that boys are more prone to being bored since they have a more outgoing temperament, while according to Shaw et al. [84], girls present a higher level of boredom because they are more controlled by parents during their free time. However, our study did not show any significant difference between males and females in the level of boredom. This finding, in line with that of Spaeth et al. [2], supports a person-context model of boredom, indicating that gender might be not directly linked to the onset of boredom, but it might moderate its behavioral and emotional consequences. 

Our study pointed out that age was also not related to the level of boredom, in line with a previous study by Sharp et al. [85], who found stable levels of boredom in adolescents aged 14 to 17.

Concerning boredom and grades, although in previous research boredom was found to impair learning and academic outcomes (e.g., [86]), no association between these variables emerged in our study. Of note is that we did not investigate adolescents’ performances in specific academic tasks, but we only considered average school marks.

The present study has several limitations that need to be mentioned. First of all, the sample considered was not balanced in terms of the proportion of males and females, and the limited number of individuals in each group prevented us from testing the MSBS for gender invariance. It is also important to mention our collective administration of the questionnaires as a limitation. Then there is the fact that, though self-report questionnaires have the advantage of being quick to administer, they can also be influenced by various factors such as individual bias, social desirability, and failure to understand a question.

In future investigations, it would be useful to apply the MSBS to a clinical population in order to identify any differences vis-à-vis a normal sample and thereby further clarify the link between boredom and psychological disorders. It will also be important to better investigate the influence of Internet use on the experience of boredom, given the hyper-connected society in which today’s adolescents live.

In conclusion, boredom in adolescence is not always entirely physiological. It may sometimes be the tip of an iceberg of severe unease and/or a risk factor for the onset of a psychiatric disorder. Weybright et al. [22] wrote that boredom is an experience that has become increasingly common among adolescents in recent years. Hence the importance of validating a tool like the MSBS for use with adolescents in order to identify excessive levels of boredom in good time, as they might conceal a general underlying discomfort. Taking a preventive approach, identifying adolescents at risk would enable us to develop specific interventions for them and thereby prevent possible maladaptive outcomes.

## Figures and Tables

**Table 1:** Mean scores on the CDI; the interpersonal sensitivity, depression, and hostility scales; and the GSI of the SCL-90-R.

Scales	*N*	Mean	Standard Deviation
CDI total score	272	13.4	7.03
Interpersonal Sensitivity	260	9.60	6.17
Depression	260	14.1	9.35
Hostility	260	5.84	4.58
Global Severity Index (GSI)	260	86.2	47.4

**Table 2:** Comparison between fit indices deriving from EFA of the models of the MSBS with one, three, four, and five factors.

Model	χ²	*df*	*p*	χ²/*df*	TLI	RMSEA	BIC
One factor	1185	377	<0.001	3.14	0.521	0.125	−667
Three factors	595	322	<0.001	1.85	0.808	0.079	−987
Four factors	485	296	<0.001	1.64	0.855	0.068	−969
Five factors	399	271	<0.001	1.47	0.892	0.058	−933

Notes: TLI, Tucker-Lewis Index; RMSEA, Root Mean Square Error of Approximation; BIC, Bayesian Information Criterion.

**Table 3:** Distribution of items and standardized saturations on their respective factors deriving from EFA of the five-factor model of the MSBS.

Item	INT	TP	HA	INA	DIS
4	1.20				
25	0.94				
29	0.73				
15	0.71				
8	0.50				
13	0.31				
24	0.31				
18		0.97			
11		0.85			
1		0.81			
26		0.80			
6		0.68			
21			0.89		
12			0.89		
5			0.82		
14			0.46		
28			0.40		
23			0.34		
2			0.31		
16				1.07	
3				0.89	
20				0.60	
10					0.88
9					0.82
7					0.58
19					0.48
22					0.42

Notes: INT, internalizing aspects; TP, time perception; HA, high arousal; INA, inattention; DIS, disengagement.

**Table 4:** Comparison between fit indexes deriving from CFA of the five-factor model.

Model	χ²	*df*	*p*	χ²/*df*	CFI	TLI	SRMR	RMSEA	AIC	BIC
Model 1	502	314	<0.001	1.60	0.884	0.871	0.065	0.066	13382	13647
Model 2	417	265	<0.001	1.57	0.901	0.888	0.062	0.065	12358	12605
Model 3	330	220	<0.001	1.50	0.920	0.908	0.059	0.061	11406	11636

Notes: Model 1, omitting items 17 and 27; Model 2, omitting items 2, 17, 23, 27; Model 3, omitting items 2, 4, 17, 23, 25, 27; CFI, Comparative Fit Index; TLI, Tucker-Lewis Index; SRMR, Standardized Root Mean Square Residual; RMSEA, Root Mean Square Error of Approximation; BIC, Bayesian Information Criterion; AIC, Akaike’s Information Criterion; BIC, Bayesian Information Criterion.

**Table 5:** Standardized and unstandardized saturations of items on their respective factors deriving from CFA of the final five-factor model of the MSBS.

			95% Confidence Interval			
Factor	Item	Unstandardized Estimate	Lower	Upper	*p*	Standardized Estimate
Internalizing Aspects (INT)	29	0.75	0.47	1.03	<0.001	0.46
	15	1.21	0.91	1.51	<0.001	0.64
	8	1.37	1.09	1.65	<0.001	0.74
	13	1.21	0.91	1.51	<0.001	0.64
	24	0.92	0.61	1.23	<0.001	0.50
Time Perception (TP)	18	1.65	1.43	1.87	<0.001	0.94
	11	1.49	1.27	1.71	<0.001	0.90
	1	1.20	0.95	1.43	<0.001	0.73
	6	1.43	1.11	1.74	<0.001	0.68
	26	1.52	1.29	1.75	<0.001	0.88
High Arousal (HA)	21	1.07	0.76	1.38	<0.001	0.59
	12	0.96	0.64	1.29	<0.001	0.52
	14	1.16	0.82	1.49	<0.001	0.59
	5	1.23	0.92	1.54	<0.001	0.66
	28	1.06	0.74	1.37	<0.001	0.58
Inattention (INA)	3	1.36	1.09	1.63	<0.001	0.79
	16	1.62	1.33	1.91	<0.001	0.84
	20	1.19	0.93	1.46	<0.001	0.70
Disengagement (DIS)	7	0.93	0.66	1.21	<0.001	0.56
	9	1.27	0.97	1.56	<0.001	0.68
	19	0.76	0.49	1.02	<0.001	0.49
	22	1.25	0.93	1.58	<0.001	0.63
	10	1.18	0.91	1.45	<0.001	0.69

**Table 6:** Descriptive indexes of the final version of the MSBS.

	INT	TP	HA	INA	DIS	TOT
M	18.3	17.3	18.2	12.4	20.3	86.5
SD	6.79	7.67	6.87	4.65	6.37	23.6
10th percentile	10	8	9	6	12	56
25th percentile	13	11	13	9	16	70
50th percentile	18	17	18	13	20	87
75th percentile	23	23	23	16	24	102
80th percentile	24	24	25	16	26	106
90th percentile	28	30	27	18	29	116

Notes: INT, internalizing aspects; TP, time perception; HA, high arousal; INA, inattention; DIS, disengagement; TOT, total MSBS score.

**Table 7:** Item-rest correlations and McDonald’s omega (ω) coefficients for the single factors and the MSBS as a whole.

Item	INT	TP	HA	INA	DIS	TOT
8	0.56					0.65
13	0.54					0.53
15	0.54					0.56
24	0.43					0.45
29	0.45					0.38
1		0.69				0.36
6		0.65				0.32
11		0.86				0.50
18		0.65				0.51
26		0.83				0.56
5			0.55			0.49
12			0.42			0.43
14			0.52			0.44
21			0.51			0.48
28			0.41			0.50
3				0.69		0.49
16				0.71		0.53
20				0.62		0.50
7					0.49	0.50
9					0.56	0.58
10					0.58	0.61
19					0.44	0.44
22					0.50	0.54
*ω*	0.74	0.92	0.72	0.82	0.75	0.90

Note: INT, internalizing aspects; TP, time perception; HA, high arousal; INA, inattention; DIS, disengagement; TOT, total MSBS score.

**Table 8:** Pearson’s r correlations between factors of the MSBS and the other tools, considering the sample as a whole.

	INT	TP	HA	INA	DIS	TOT MSBS
TOT CDI	0.68	0.12	0.51	0.38	0.42	0.57
INT. SENS	0.58	0.03	0.46	0.27	0.25	0.43
DEP	0.69	0.09	0.56	0.32	0.35	0.55
HOS	0.32	-0.01	0.40	0.26	0.28	0.33
GSI	0.63	0.07	0.56	0.34	0.35	0.53

Notes: INT, internalizing aspects; TP, time perception; HA, high arousal; INA, inattention; DIS, disengagement; TOT MSBS, total MSBS score; TOT CDI, total CDI score; INT. SENS, interpersonal sensitivity; DEP, depression; HOS, hostility; GSI, global severity index.

## Data Availability

Data presented in this study are available on reasonable request to the corresponding author. Data are not publicly available because they report private information about participants.

## Appendix A

Multidimensional State Boredom Scale (MSBS)—Italian Adolescent Version (English translation)

Instructions: for each of the claims, please indicate how you see yourself and your life at the present time, even if these feelings differ from those you have normally. Choose one of the following numbers:

1 = strongly disagree; 2 = disagree; 3 = slightly disagree; 4 = neither agree nor disagree; 5 = slightly agree; 6 = agree; 7 = strongly agree.

1. Time is passing more slowly than usual

2. I’m easily distracted

3. Everything seems to irritate me at the moment

4. I’d like time to pass more quickly

5. Everything seems repetitive and routine

6. I feel low

7. I feel like I’m being obliged to do things of no value to me

8. I feel bored

9. Time passes too slowly

10. I’m more moody than usual

11. I’m undecided or unsure about how to proceed

12. I feel irritable

13. I feel empty

14. I have trouble concentrating

15. Time passes slowly

16. I’d like to do something more exciting

17. My attention span is shorter than usual

18. I feel impatient

19. I’m wasting time that it would be better to spend doing something else

20. I want something to happen, but I don’t know exactly what

21. Right now it seems like time is passing slowly

22. I feel as if I’m just sitting waiting for something to happen

23. It feels like there’s nobody around me I can talk to

Scoring instructions: sum the points per item to obtain the score of each scale.

Time perception: items 1, 4, 9, 15, 21; Inattention: items 2, 14, 17; High arousal: items 3, 10, 12, 18, 22; Disengagement: items 5, 7, 8, 16, 19; Internalizing aspects: items 6, 11, 13, 20, 23; Total score: all items.

## Appendix B

Multidimensional State Boredom Scale (MSBS)—Italian Adolescent Version

Istruzioni: Per favore, rispondi a ognuna delle affermazioni indicando il modo in cui senti te stesso e la tua vita in questo momento, anche se si tratta di sensazioni che differiscono da come ti senti di solito. Scegli uno fra i seguenti numeri:

1 = Fortemente in disaccordo; 2 = In disaccordo; 3 = Un po’ in disaccordo; 4 = Né d’accordo né in disaccordo; 5 = Un po’ d’accordo; 6 = D’accordo; 7 = Fortemente d’accordo.

1. Il tempo sta passando più lentamente del solito

2. Mi distraggo facilmente

3. Tutto sembra irritarmi in questo momento

4. Vorrei che il tempo scorresse più velocemente

5. Tutto mi sembra ripetitivo e routinario

6. Mi sento giù

7. Mi sembra di essere costretto a fare cose che non hanno alcun valore per me

8. Mi sento annoiato/a

9. Il tempo scorre troppo lentamente

10. Sono più lunatico/a del solito

11. Sono indeciso/a o insicuro/a su come procedere

12. Mi sento agitato/a

13. Mi sento vuoto/a

14. Ho difficoltà a concentrarmi

15. Il tempo scorre lentamente

16. Vorrei fare qualcosa di più eccitante

17. La mia capacità di attenzione è più breve del solito

18. Mi sento impaziente

19. Sto sprecando tempo che sarebbe meglio impegnato in qualcos’altro

20. Voglio che succeda qualcosa, ma non so di preciso cosa

21. In questo momento sembra che il tempo passi lentamente

22. Mi sento come se fossi seduto/a ad aspettare che accada qualcosa

23. Sembra che non ci sia nessuno attorno a me con cui parlare
